# Generative adversarial networks in EEG analysis: an overview

**DOI:** 10.1186/s12984-023-01169-w

**Published:** 2023-04-11

**Authors:** Ahmed G. Habashi, Ahmed M. Azab, Seif Eldawlatly, Gamal M. Aly

**Affiliations:** 1 Computer and Systems Engineering Department, Faculty of Engineering, Ain Shams University, 1 El-Sarayat St., Abbassia, Cairo, Egypt; 2 Biomedical Engineering Department, Technical Research Center, Cairo, Egypt; 3 Computer Science and Engineering Department, The American University in Cairo, Cairo, Egypt

**Keywords:** EEG, GAN, P300, Motor imagery, Emotion recognition, Epilepsy

## Abstract

Electroencephalogram (EEG) signals have been utilized in a variety of medical as well as engineering applications. However, one of the challenges associated with recording EEG data is the difficulty of recording large amounts of data. Consequently, data augmentation is a potential solution to overcome this challenge in which the objective is to increase the amount of data. Inspired by the success of Generative Adversarial Networks (GANs) in image processing applications, generating artificial EEG data from the limited recorded data using GANs has seen recent success. This article provides an overview of various techniques and approaches of GANs for augmenting EEG signals. We focus on the utility of GANs in different applications including Brain-Computer Interface (BCI) paradigms such as motor imagery and P300-based systems, in addition to emotion recognition, epileptic seizures detection and prediction, and various other applications. We address in this article how GANs have been used in each study, the impact of using GANs on the model performance, the limitations of each algorithm, and future possibilities for developing new algorithms. We emphasize the utility of GANs in augmenting the limited EEG data typically available in the studied applications.

## Introduction

Electroencephalography (EEG) is widely accepted as one of the most popular methods of non-invasive techniques for recording brain activity that can be used in cognitive studies, different clinical applications, and brain-computer interfaces (BCIs) [1]. In fact, EEG recording plays a crucial role in several domains where it directly measures the aggregated neural activity in addition to being an easy portable method for different clinical uses. Furthermore, advances in machine learning and other recent technologies such as wireless recording have led to more interest in EEG-based BCI approaches, which could enhance the quality of life of people with disabilities. EEG recording is considered inexpensive compared to other non-invasive brain signal recordings technologies such as functional magnetic resonance imaging (fMRI), magnetoencephalography (MEG), and near-infrared Spectroscopy (NIRS) [1,2].

Unfortunately, there are different circumstances where EEG data could not be fully utilized due to data-related problems such as corruption, scarcity, noise, and muscle artifacts [2]. In addition, EEG analysis faces challenges and suffers from limitations due to its low signal-to-noise ratio (SNR) [3]. EEG is also considered a non-stationary signal as it varies from one subject to another, and even from one recording session to another for the same subject [4,5]. On the other hand, machine learning models, such as deep neural networks, which are being increasingly used in analyzing EEG signals require large training sets to achieve the accepted classification accuracy. Thus, a large amount of data needs to be available to effectively train a robust system that can recognize different brain patterns. However, it is time-consuming and uncomfortable to conduct long calibration sessions especially when the involved subjects are patients, children, or the elderly. Due to these limitations, machine learning classifiers trained on EEG datasets can hardly keep their performance accepted, especially with limited amounts of data [6].

**Fig. 1:**
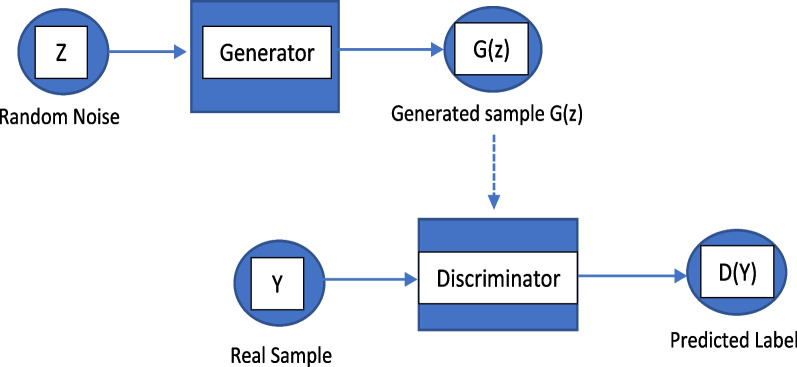
GAN architecture

In such a sense, there is a great need to augment EEG signals with data that bear a resemblance to recorded data to increase the size of the data. Although Generative Adversarial Networks (GANs) were originally proposed as deep learning models for image generation, these models could represent the potential solution for EEG data augmentation (DA). A GAN mainly comprises two opponent networks: the generator network and the discriminator one [7]. Figure 1 illustrates a simplified schematic of a GAN. The generator is used to capture the distribution of the training data and tries to generate additional samples that are not recognized as fake (i.e., not part of the original data) by the discriminator. On the other hand, the discriminator acts as a binary classification model that decides whether the input data originates from real data or not. Ultimately, this competition between the generator and discriminator networks leads to the generation of artificial data of high quality that resembles the original input data. Although GANs have been investigated in many image processing and computer vision applications [8], their utility in augmenting EEG data is not fully explored. In fact, there is a lack of review in comparing these GAN algorithms when applied to EEG signal analysis. Therefore, we aim here to provide a comprehensive overview of the state-of-the-art GAN algorithms in application to EEG signal analysis.

The organization of this article is as follows: We first present an overview of GANs and their most common types in Sects. "Selection criteria" and "GANs overview". In Sect. "GANs for EEG tasks", we review the utilization of GANs in each of the following main EEG analysis applications: Motor imagery, P300, RSPV, emotion recognition, and epilepsy, in addition to various other paradigms. The analysis and discussion of the reviewed papers are provided in Sect. "Discussion". Finally, a conclusion is provided in Sect. "Conclusion".

## Selection criteria

**Fig. 2:**
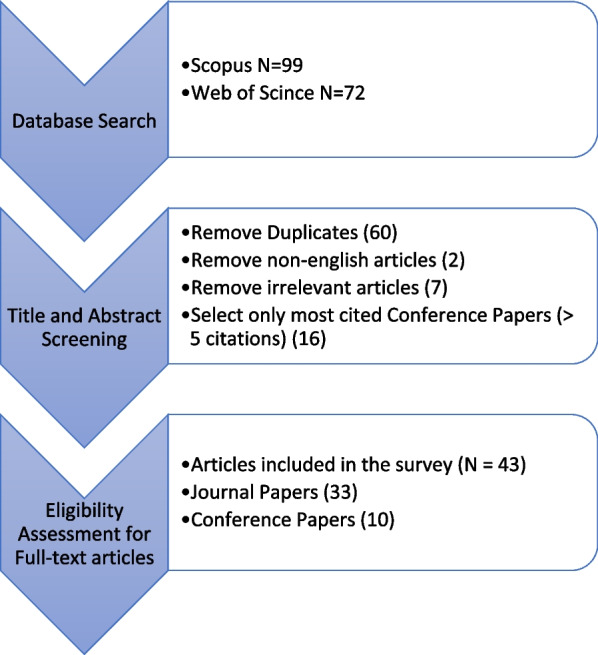
Paper selection criteria

The main purpose of this article is to survey different GAN methods that have been used in different EEG experiments emphasizing how these algorithms aided in solving problems of various EEG-based tasks. The literature review has been conducted as shown in Fig. 2 across two main well-known databases: Web of Science and Scopus, on December 8, 2021. The key terms that were included in the search are [(Generative Adversarial Networks) AND (Electroencephalography)] OR [(GANs) AND (EEG)], and other similar entries. The primary search yielded a total of 171 articles published between 2015 and 2021. These articles were first scanned based on their titles and abstracts to ensure that the search strategy accurately detected the targeted articles. Hence, articles irrelevant to the topic area, non-English articles, duplicated articles, and conference proceeding papers (except the most cited ones), were excluded. As a result of this selection criteria, a total of 43 (articles, most cited conference proceeding) papers have been surveyed to complete the current study.

After identifying the papers of interest, data were extracted separately for each paper to cover title, authors, year of publication, the main purpose of the paper, the datasets used, the type of GAN used, GANs outcomes, evaluation method used, and where available, GANs effect compared to other examined algorithms.

## GANs overview

### Basic concepts

Generative Adversarial Networks (GANs) consist of two opposing networks, the generator $\left(G\right)$ and the discriminator $(D)$ complete each other to generate data as close as possible to the real data [7]. The *G* network always tries to capture the signal’s distribution and produces real-like data from a random noise vector input (*z*). Meanwhile, the *D*, a binary classifier, evaluates the generator output and distinguishes fake samples (*G*(*z*)) from real ones. Both networks are trained in parallel where the competition between the two networks ultimately results in the generation of artificial high-quality data. While the training process of the $G$ network aims to maximize the probability that *D* classifies generated samples as “real” ones, the opponent training of the $D$ network seeks to maximize the probability of detecting “real” data from “fake” data. In other words, the two models try to minimize an adversarial loss function by playing the following minimax game [7]

1 $${\mathit{min}}_{G}\,{max}_{D}V\left(D,G\right)= {E}_{x\in {P}_{r}}\left[\mathit{log}D\left(x\right)\right]+{E}_{z\in P}[log(1-D(G\left(z\right)))]$$

where *E* is the expected value, ${P}_{r}$ describes real data distribution, *z* represents the random noise vector from the latent space of the simple noise distribution *P*, *G*(*z*) represents the data generated by *G,* and *D*(*x*) is the probability that *x* is a real data. The optimization process in (1) is equivalent to minimizing the Jensen-Shannon (JS) divergence [7].

### Main variant architectures of GANs

Several studies have investigated the problems that appeared with the first versions of GANs such as mode collapse, where *G* is only able to generate one or a few subsets of different outcomes, or modes [9]. Also, one of the most common causes of training instability when the vanishing gradient occurs during the training process of *G* is that the discriminator would be no longer deceived by the fake samples’ output [10]. This happens as *D* of the original GAN usually tends to rapidly reach optimality and the JS divergence (between the distribution of real data and the generated ones) does not converge leading to learning failure [11].

#### The conditional GANs

Mirza and Osindero introduced conditional GANs (cGANs) [12 13]. The main concept of cGAN is that both networks have inputs of conditioning data. The generator is fed with random noise vectors $(z)$ appended with additional information (*y*) that is typically the condition labels, also the labels are combined into the discriminators. The optimization formulation of cGAN can be defined as:

2 $${min}_{G}\,{max}_{D}V\left(D,G\right)\,= {E}_{x\in {P}_{r}}\left[\mathit{log}D\left(x,y\right)\right]+{E}_{z\in P}[log(1-D(G\left(z,y\right),y))]$$

where *y* is the label of the corresponding *x*. In addition, cGAN training follows the same procedure as GAN training and with the same measure of generated samples’ JS divergence. Therefore, cGAN still faces the same problems of mode collapse and unstable training.

#### The deep convolutional GANs (DCGANs)

Deep convolutional GAN (DCGAN) was one of the early modifications of GANs that utilized deep convolutional neural networks (CNN) for both the generator and the discriminator for better training [14]. DCGAN implementation is based on the loss function in Eq. 1. In DCGAN, instead of the pooling layer, the discriminator uses a stride convolution layer, and the generator uses the transpose convolution (fractional-stride convolution). In addition, the fully connected classification layer, which is a subsequent layer to the convolutional layer in the original CNN, is removed. Instead, batch normalization is used with each convolutional layer which supports the gradient flow [15].

#### The Wasserstein GAN (WGAN)

The Wasserstein GAN (WGAN) was introduced in [16] in addition to the improved version proposed by Arjovsky et al. where they replaced the discriminator network with a critic (C) [17]. This critic measures the distance between the real and model distributions depending on the Earth Mover’s distance (*EM* (*p*, *q*)), or Wasserstein-1, which is a metric of the minimum cost for moving distribution elements (earth mass) to transform a distribution *q* to distribution *p* (cost = mass × transport distance). The original GAN algorithm tries to minimize the JS divergence between the real data distribution ${P}_{r}$ and fake data distribution ${P}_{g}$. For WGAN, EM will not eventually act as a binary classifier only and decide if a sample is fake or not, but also it will be able to determine how real or fake the produced sample is as a continuous regressive output. Consequently, the critic will converge to a linear function with the right training. In addition, the gradients will be acceptable, the process will avoid saturation, and could solve the problem of mode collapse. The Wasserstein GAN loss function is obtained by the Kantorovich-Rubinstein duality [17 18]

3 $${\mathit{min}}_{G}\,{max}_{C\in F} {E}_{x\in {P}_{r}}\left[D(x)\right]-{E}_{\widetilde{x}\in {P}_{g}}[D(\widetilde{x})]$$

where *F* is the set of 1-Lipschitz functions, ${P}_{r}$ the real distribution, ${P}_{g}$ the model distribution defined by $\widetilde{\mathrm{x}}= G(z), z \in p(z)$, and *z* is the random noise. If *C* is optimal, minimizing the value function with respect to *G* minimizes EM (${P}_{r}$,${P}_{g}$). Although WGAN helps to solve the problems of training instability, enforcing the Lipschitz continuity by clipping the weights of the discriminator to an interval [− *c*, *c*] is sometimes fruitless [19 20].

#### The Wasserstein GAN-Gradient Penalty (WGAN-GP)

Because of WGAN weight clipping, convergence failure and poor generation of samples are the most common problems of WGAN [9]. Thus, penalizing the gradient norm of the discriminator regarding its input was proposed as a potential solution. This method is called Wasserstein GAN-Gradient Penalty (WGAN-GP). The experimental findings demonstrated that WGAN-GP achieves, with limited hyperparameter tuning, stable training of different GAN architectures. The objective function of WGAN-GP is:

4 $${min}_{G}\,{max}_{D}V\left(D,G\right)\,= {E}_{x\in {P}_{r}}\left[D(x)\right]-{E}_{\widetilde{x}\in {P}_{g}}\left[D\left(\widetilde{x}\right)\right]- \lambda {E}_{\widehat{x}\epsilon {P}_{\widehat{x}}}[(||{\nabla }_{\widehat{x}}D\left(\widehat{x}\right){||}_{2}-1{)}^{2}]$$

where λ is the gradient penalty coefficient and $\widehat{x}$ is sampling along straight lines between the real data distribution ${P}_{r}$ and the generated data distribution ${P}_{g}$

5 $$\widehat{x} = \varepsilon + \left(1 - \varepsilon \right)\widetilde{x}, \varepsilon \in uniform \, \left[0, 1\right], x\in {P}_{r}, \widetilde{x}\in {P}_{g}$$

## GANs for EEG tasks

In this paper, the surveyed papers have been classified into 5 main groups: motor imagery, RSVP and P300, emotion recognition, epilepsy studies, and other EEG applications.

### Motor imagery

Motor Imagery (MI) is the activation of motor-related brain regions because of imagining a specific body part’s movement [21]. The decoding of the MI EEG signals is considered one of the main pillars of BCI studies. Through the years, MI has proven its crucial role in providing means of communication and control for people with movement impairments paraplegia and stroke patients without relying on muscle activity [22]. MI-based BCIs do not require any other external stimuli. Identifying intended movement in MI-based BCIs is based on recognizing the decrease and increase of oscillatory activity in certain bands, which is induced by imagined motion, termed event-related desynchronization, and synchronization (ERD and ERS), respectively [23]. Whilst movement imagination generates ERD in the mu EEG band (8–12 Hz) and beta EEG band (18–26 Hz), relaxation causes ERS [24]. The right/left-hand movement could be decoded from the patterns of ERS, and ERD evoked in the C3, C4, and Cz EEG electrodes as defined by the standard positions of the International 10–20 system. The generated ERD/ERS from motor imagery has the same topography and spectral performance as the real movements’ patterns [24]. However, MI could be considered a skill that requires learning and training. Unfortunately, long training sessions (20–30 min) are required to calibrate MI-based BCI systems to achieve an accepted performance [25]. Thus, GANs could play a crucial role by augmenting the limited available data for training to compensate for the need for long calibration sessions. We review here the most recent research demonstrating the use of GANs in data augmentation (DA) to improve MI-based BCIs performance. Table 1 summarizes all the reviewed articles with the type of GAN used in MI tasks.

**Table 1:** Reviewed papers that used GANs in motor imagery tasks

Study	Purpose	Dataset	GAN Type	Evaluation metrics	Results (with GAN)	
Abdelfattah et al., 2018 [26]	Enhance the model classification performance	PhysioNet	RGAN	Reconstruction accuracy	Reconstruction: • + 19.9% (w.r.t VAE) • + 34.8% (w.r.t AE)	
				Classification accuracy	Classification (25% dataset): • DNN + 36.1% • SVM + 14.1% • RFT + 13.1% Classification (50% dataset): • DNN + 39.1% • SVM + 12.8% • RFT + 12.6%	
Hartmann et al., 2018 [27]	Achieve stabilization of the training	Private dataset	WGANs	• Classification accuracy • IS • FID • ED • SWD	• 91.25% • 1.363 • 9.523 • − 0.056 • 0.078	
Corley and Huang 2018 [28]	Produce high spatial resolution EEG data from low-resolution samples	Berlin BCI Competition III, Dataset V	WGANs	• Classification accuracy	Scale 2: 3.87 < HR Scale 4: 5.75 < HR	
				• MSE • MAE	Scale 2: • − 37,497,940 • − 3885.34	Scale 4: • − 72,991,320 • − 6385.61
Fahimi, Zhang et al., 2019 [29]	Enhance the model classification performance	A public dataset collected by [40]	DCGANs	Classification accuracy	NA	
Fahimi, Dosen et al., 2021 [30]	Enhance the model classification performance	Private dataset + BCI competition III, Dataset IVa	DCGANs	Classification accuracy	• Diverted attention: + 7.32% (p < 0.01) • Focused attention: + 5.45% (p < 0.01) • IVa: + 3.57% (p < 0.02)	
Li and Yu 2020 [32]	Enhance the model classification performance	BCI competition IV, Dataset 2b	cWGAN-GP	Classification accuracy	(w.r.t raw data) • Shallow + 1.65% • Deep4 + 2.89%	
Debie et al., 2020 [33]	Protect EEG brain signals against illegal disclosure	BCI competition IV, Dataset 2a	GAN with differential privacy	Classification accuracy	NP-GAN (max:150 trial) • SVM + 5.74% • RF + 3.43% • LDA + 6.39% • LR + 9.54% PP-GAN (max: 50 trial) • SVM − 1.05% • RF + 0.36% • LDA − 0.19% • LR + 0.18%	
Zhang et al., 2020 [15]	Avoid overfitting and enhance the model classification performance	BCI competition IV (datasets 1 + 2b)	CNN-DCGAN	Classification accuracy	• D1: + 8.7% (1:3) • D2b: + 12.6% (1:3)	
				kappa value	• D1: + 0.1622% • D2b: + 0.1981%	
Luo et al., 2020 [35]	Reconstruct EEG signal with high sampling rates and sensitivity	Private dataset + Lucid et al., 2014 + BCI competition IV, dataset 2a	WGAN + (TSF-MSE) loss function	Classification accuracy	• MI: + 2.03% • AO: + 4.1% • GAL: + 4.11%	
				Reconstruction accuracy	• MI: + 3.2% • AO: + 4.5% • GAL: + 5.38%	
Yang et al., 2021 [38]	Address the challenge of insufficient MI data	Private dataset + BCI competition IV, Dataset 2a	cVAE- GAN	Classification accuracy	86.14% D1 mean ~ + 4% D2 mean ~ + 1.5%	
				• IS • FID • SWD	w.r.t Real • − 0.121 • + 11.364 • + 0.067	
Xie et al., 2021 [21]	Address the challenge of insufficient MI data	BCI competition IV, datasets 2a + 2b	LGANs (augmentation) + MoCNN (classification) + attention network	Classification accuracy	D1 (w.r.t raw data) • LGAN + 8.23% • Att-LAGN + 9.34% D2 (w.r.t raw data) • Att-LAGN: + 5.64% − 6.6%	
Xu et al., 2021 [39]	Enhance the model classification performance	Private dataset	CycleGAN	Classification accuracy	+ 18.3%	

One of the earliest studies is the study by Abdelfattah et al. which introduced a recurrent GAN (RGAN) model for generating synthesized EEG data to increase the dataset size [26]. Recurrent neural networks (RNNs) were employed in the generator component, while the discriminator consisted of three hidden fully connected layers trained in a supervised manner to minimize the mean square error (MSE). The performance of the proposed RGAN was evaluated for EEG motor movement/imagery events (eye close (EC)—eye open (EO)—open left fist (OLF)—and open right fist (ORF)) by using three different classification models: deep feed-forward neural network (DNN), support vector machine (SVM) and random forest tree (RFT). Moreover, the performance of the RGAN-generated data was evaluated against two other DA methods (autoencoders (AE) and variational autoencoders (VAE)). The results demonstrated that the RGAN model improved the accuracy by an average of 34.8% and 19.9% relative to AE and VA, respectively. Furthermore, employing RGAN for augmentation using only 25% of the available dataset showed that the DNN performance was remarkably improved by 36% compared to its performance without RGAN-generated data. In addition, DNN trained using RGAN-generated data from 25% of the training dataset was 13.8% and 7.1% higher than SVM and RFT, respectively. Finally, using 50% of the dataset and employing RGAN for augmentation, the performance of the DNN significantly outperformed the SVM and RFT by an average of 21% and 14%, respectively.

The use of Wasserstein GANs was employed in MI data analysis by Hartmann et al. for generating realistic samples of EEG data [27]. They adopted a modification of WGAN to achieve more training stability as GANs training usually suffered from vanishing gradients during optimizing the JS Divergence [7]. Thus, the modified WGAN solved this issue by decreasing the Wasserstein distance and depending on the gradual smoothing of the gradient constraint. In addition, the one-sided gradient penalty term was adopted for the discriminator or critic instead of the two-sided penalty one. The authors used multiple metrics to evaluate the proposed model such as Inception Score (IS)—Fréchet inception distance (FID)—Sliced Wasserstein distance (SWD)—Euclidean Distance (ED). However, IS did not provide meaningful information about the quality of generated signals. Thus, they used (FID, SWD, and ED) together as they held sufficient information on the model properties. The best IS achieved was 1.363 using a structure of stride convolution for downsampling and linear interpolation. Also, nearest neighbor upsampling with average pooling attained the best FID and ED. Eventually, the stride convolution downsampling with cubic interpolation achieved the lowest SWD of 0.078.

Corley et al. employed WGAN to generate EEG super-resolution (SR) signals for MI [28]. They used WGANs for generating channel-based upsampled data to interpolate various missing channels. The authors noted that WGAN was more stable during training compared to the original GAN algorithm and the EEG SR task was extremely sensitive to the loss function components. Moreover, there was a remarkable improvement in simultaneously reconstructing missing EEG signals at high resolution by using the proposed WGAN method. Finally, the classification of SR data had an accuracy with minimal loss compared to baseline signals, with a reduction of 4% and 9% for scale factors of 2 and 4, respectively. Furthermore, the WGAN architecture achieved an obvious improvement in terms of Mean Square Error (MSE) and Mean Absolute Error (MAE).

In [29], Fahimi et al. proposed another framework to generate artificial EEG by using deep convolutional GANs (DCGAN). DCGANs were trained on raw MI data and then the trained generator produced synthetic EEG data from the random noise input. Investigating the similarity between the generated and the real EEG data in time and frequency domains showed that the generated EEG signals clearly had the temporal, spectral, and spatial characteristics of real EEG. In another follow-up study by members of the same group [30], the use of DCGANs was explored to achieve a classifier performance improvement. The proposed DCGANs framework is powered by a subject-specific conditioning vector and modified objective and loss functions. The comparison between the proposed DCGANs framework and two other DA methods; VAE and Segmentation and Recombination (S&R); was performed using MI data of focused attention and diverted attention conditions. The quality, diversity, and reality of the generated samples were evaluated using three tests (GAN test -KL divergence- 2D visualization using t-SNE spectrogram [31] and temporal distribution). The end-to-end DCNN, VAE, and conditional DCGANs were implemented in Python 3.6 with Keras 2.1.2 and Tensorflow 1.2.1. The results showed that using the proposed DCGANs-based framework outperformed S&R/VAE, especially in the diverted condition. The average accuracy of the DCGANs was the highest with 85.54% for focused conditions and 80.36% for diverted ones. Meanwhile, DCGANs attained a total improvement of 5.45% for focused attention and 7.32% for diverted attention as validated by leaving one subject-out (LOSO) classification in comparison to the deep convolutional neural network (DCNN). In addition, the testing of the proposed framework on dataset IVa from BCI competition III increased the accuracy by 3.57%. It is worth mentioning that the DCGAN algorithm did not suffer from training instability as both networks gradually converged, especially under the diverted condition.

In [32], Li et al. proposed a Conditional WGAN-GP (cWGAN-GP) to synthesize EEG data. They utilized two known available CNN architectures for a motor imagery task (the Deep4 and the Shallow from Braindecode2). The comparison between the classification task model trained with and without an augmented dataset was held. As a result, the classification accuracy showed an obvious improvement for the Deep4 model and the Shallow model. Using cWGAN, applied to Shallow improved the classification accuracy from 72.97% by 1.65%, while Deep4 improvement was 2.89%. Furthermore, it was clear that whenever the size of generated EEG data is less than the original dataset size, the classification improvement accuracy was more obvious.

With a new vision for employing GAN algorithms, Debie et al. proposed a privacy-preserving GAN method to generate and effectively classify EEG data [33]. The proposed approach was evaluated using benchmark EEG data of the MI set [34]. The differential privacy concept was introduced by Cynthia Dwork to achieve personal privacy by eliminating sensitive data from a database. They preserve the data privacy by generating real-like EEG data instead of sensitive recorded data from subjects that could reveal the identity of their participants during the model training. Two GANs were trained with the adoption of the differentially private stochastic gradient descent (DP-SGD) approach during synthesizing EEG data; a non-private GAN (NP-GAN) and a private GAN (PP-GAN) trained under differential privacy. The proposed approach aimed to reduce the individual’s effect during training on the gradient computations. Consequently, a specific subject’s statistical distribution would not be learned by the NN model. SVM, RF, linear discriminant analysis (LDA), and logistic regression (LR) classifiers were trained to recognize left from right-hand movement. Consequently, the results for all subjects had a similar pattern where the generator loss gradually declines, whereas the discriminator loss rises to equilibrium (both losses are very close to each other). In addition to that, setting the noise multiplier, a controller for the added noise, to 1.4 or higher produced a remarkable deterioration in classification performance for three classifiers (SVM, RF, and LDA). Interestingly, augmenting training data with up to 150 artificially generated data increased the classification accuracy for all three classifiers, but 200 artificial samples had the worst results. It is therefore clear to say that the ratio of generated to raw data could be tuned to increase the classification performance of different classifiers.

In [35], Luo et al. adopted a high sampling sensitivity EEG reconstruction algorithm from low sampling signals based on WGAN and a temporal-spatial-frequency (TSF-MSE) loss function. In this method, the discrepancy between different sampling rates, sensitivities, and a TSF loss function determines the difference between EEG signals in the feature domain. The WGAN architecture comprised three main parts: the deep generator, the TSF-MSE loss calculator, and the discriminator. TSF-MSE-based loss function generates signals by computing the MSE from the following features: temporal MSE between time steps (as a typical MSE), spatial MSE between channels, and frequency MSE between signal batches. In other words, the proposed algorithm depends on extracting not only time-sampling features but also spatial and frequency features using both common spatial patterns (CSP) and power spectral density (PSD) with WGAN. During this study, three different EEG datasets were used; Action Observation (AO) dataset [36], Grasp and Lift (GAL) dataset [37], and the MI dataset from BCI competition IV dataset 2a. Both GAN/WGAN frameworks were implemented in Python 2.7 with the Tensorflow 1.8 library. The reconstruction results using WGAN of the same sensitivity showed its outperformance, however, the GAN algorithm was better than the WGAN for reconstructions of different sensitivity. In addition, the quantitative analysis asserted that the WGAN framework had a higher classification accuracy with more reliable statistical properties due to more discriminant patterns. Besides, the TSF-MSE-based GAN/WGAN models produced fewer losses than the temporal MSE, frequency MSE, and spatial MSE-based GAN/WGAN models. Overall, WGAN achieved the best classification accuracy with 67.67%, 73.89%, and 64.01% for the AO dataset, GAL dataset, and MI dataset, respectively, with a total enhancement of 4.1%, 4.11%, 2.03% for the same datasets, respectively.

Also in 2020, Zhang et al. conducted research on DA methods used for EEG signals based on the FID and classification of MI data [15]. In this study, the DCGAN framework was applied to the obtained spectrograms of MI data that are subsequently classified by a CNN model to verify the classification performance after augmentation. In addition, DCGAN was compared to other DA methods including Geometric Transformation (GT), Noise Addition (NA), AE, and VAE. FID was used to evaluate the quality of the generated data and the classification accuracy. Using both benchmark BCI Competition IV (2b and 1) datasets, CNN-DCGAN achieved an average classification accuracy enhancement of 12.6% and 8.7% higher than the baseline. In turn, the proposed CNN-DCGAN model outperformed the best classification method in previously mentioned DA methods as it exceeded the average accuracy of both VAE and AE by 5% for dataset 1. In addition, the accuracy of DCGAN was higher than that of VAE and AE by 5.6% and 10%, respectively, for the classification accuracy of dataset 2b.

Yang et al. proposed a novel combination of a conditional VAE network (cVAE) with GAN for feature sub-space generation of MI-EEG brain signals. [38]. In this model, an encoder network learned the temporal and spectral features of real MI samples and mapped them to a latent representation *z* through a CNN. The study posits that this kind of combination led to more robust training with faster convergence as it took advantage of both statistic and pairwise features. IS, FID, and SWD were used as evaluation metrics. The implementation of this model was performed using Python and Keras API. Results demonstrated that the test accuracy of the classifier was 86.14% which was remarkably higher than the accuracy achieved without DA for almost all subjects. Furthermore, cVAE-GAN had the best performance in IS and SWD metrics. Although CNN outperformed CVAE-GAN in FID, it had worse values in other metrics.

Xie et al. suggested another combination of long short-term memory with GAN (LGANs) and multi-output CNN (MoCNN) for MI classification [21]. The generator of this model consisted of a fully connected layer with four convolutional layers. Meanwhile, the discriminator had three convolutional layers, one LSTM, and one fully connected layer. Then, the MoCNN, which uses the feature information that is extracted from each layer of the convolutional layer, was proposed to improve the classification performance. For enhancing model performance, an attention network was used with the generator to enable the generator to focus on the key feature information of MI data and the channels interconnection and sampling points. The results showed the outperformance of the proposed model compared to the other GAN models used in the same study (cGAN – WGAN – infoGAN – lsGAN – softmaxGAN – lGAN – AttGAN without LSTM – AttGAN). While the proposed model results for the BCI competition 2a dataset achieved an average accuracy of 83.99%, all other GANs models only attained lower accuracies from 59.79% by cGAN to 82.88% by the LGAN. Meanwhile, without data augmentation, the MoCNN classification model only had a classification accuracy of 74.65%. Moreover, when the proposed method was applied to the BCI competition 2b dataset, a significant performance was observed with an average accuracy of 94.31% which was higher than WGAN, cGAN, and even LGAN.

In [39], CycleGAN was used to generate MI data for stroke patients where EEG data was converted to EEG-topography images that had both spatial and spectral features of the EEG. The study adopted S-transform to effectively evaluate ERD/ERS of the EEG, in turn, they could classify different types of MI tasks. The EEG spectral topographies of healthy subjects were then used for CycleGAN training. Finally, a seven-layer CNN network and SVM were alternatively employed for classification. The data from five subjects were used for testing the algorithm where CNN outperformed SVM for all subjects in terms of classification accuracy. In addition, it was noticed that a significant improvement in classification accuracy, compared to the raw training data, occurred as the generated EEG data was added to the training set by 1-time of dataset samples, 4 times, and 5 times. The accuracy with one of the subjects by CycleGAN data augmentation reached 78.3% compared to 60% for the original data.

### P300 and RSVP

P300 and Rapid Serial Visual Presentation (RSVP) represent two other main paradigms of BCI experiments [41]. P300 evoked potentials represent one dominant component of Event-Related Potentials (ERP). Mainly, the P300 signal appears as a positive wave in the EEG due to irregular auditory, visual, or somatosensory stimuli [42], that are evoked 300–400 ms after attendance to a rare target stimulus that occurs among several frequent stimuli [43]. P300 recognition has been utilized to develop significant communication tools and devices for patients who have motor neuron diseases. P300-based BCIs are able to provide such patients with affordable, mobile, and non-invasive communication devices that would enhance their quality of life. Despite the progress P300-based BCIs have had, detecting P300 signals and their interpretation faces some challenges. For instance, such waves are more likely to be high-dimensional with poor signal-to-noise-ratio [44]. In addition, P300 signals have been shown to be non-stationary with high inter-subject variability [42].

One of the most common experiments that utilize P300 signals is RSVP. The main concept of RSVP could be easily clarified with an example of an experiment for examining visual attention where stimuli are presented frequently to participants. Then, participants try to select a specific target from the presented ones and ERPs or P300 signals could be detected in the generated target EEG samples [45]. Therefore, this section will focus on the surveyed papers that employed GANs techniques with P300 signals and RSVP experiments. Table 2 shows the proposed trials to augment these waves by using several methods of GANs to alleviate the mentioned issues.

**Table 2:** Reviewed papers that used GANs in P300 and RSPV tasks

Study	Purpose	Dataset	GAN type	Evaluation metrics	Results (with GAN)
Ming et al., 2019 [46]	Overcome challenges for Bio-signals as intra- and cross-subject variance	MNIST + private dataset (driving)	SAN	Classification accuracy	+ 1%
Panwar et al., 2019 [48]	Address training instability and frequency artifacts	BCIT X2	cWGAN-GP	Classifier AUC	• Same subject evaluation + 3.28% • Cross-subject evaluation + 5.18%
Panwar et al., 2020 [45]	Generate EEG data to improve the classification performance of cognitive events	BCIT X2	WGAN-GP + CC-WGAN-GP	Classifier AUC	CC-WGAN-GP: + 5.83%
Kunanbayev et al., 2021 [42]	Overcome the scarcity problem of training for robust classifier model	P. Arico et al.	DCGAN + WGAN-GP	Classification accuracy	+ 2%: + 4%

To reduce variations of EEG signals from different sessions or different subjects that lead to poor generalization of the trained models, a subject adaptation network (SAN) based on GANs was proposed in [46]. Ming et al. designed SAN which sampled the real data as generator input instead of the noise source, which enables the generator to learn how to align multiple source distributions to a consistent one to fool the discriminator. In addition, instead of feeding the real samples as base data to the discriminator, it sampled the generated distribution. To evaluate the SAN algorithm, the MNIST dataset, as a multimodal distribution, was firstly utilized. Afterward, a recorded EEG dataset of visual evoked potential (VEP) oddball task experiment that was based on the P300 was performed. Also, a private driving EEG dataset, where EEG signals were recorded during a driving task, was exploited to prove the network’s ability to sample selection, particularly from the intra-subject variance perspective. A comparison between SAN results with both the SVM method and EEGNet [47], compact CNN for EEG-based BCIs, that led to equivalent results was included. Results demonstrated that the SAN model is slightly better than other models with an average classification accuracy of 81.5%. Overall, the proposed model demonstrated its practicability and effectiveness with various datasets.

In [48], a conditional WGAN-GP was proposed for generating EEG data of different cognitive events with minimum high-frequency artifacts. Single-channel EEG training data was used from the RSVP experiment. The proposed architecture was based on bicubic interpolation upsampling of the input dimension and a deconvolution layer with bilinear weight initialization. The two-step upsampling method was used to avoid frequency artifacts and made GAN training more stable. For mode collapse avoidance, they utilized class labels in both the generator and discriminator. Then for evaluating the generated samples, the authors determined the log-likelihood of Gaussian mixture models of the real samples. Although the trained classifier got only 75% AUC, visual inspection, and Log-Likelihood distance from Gaussian Mixture Models (GMMs) showed that the generated samples had an acceptable quality and could capture the main characteristic of the real samples. Moreover, it was noticed that the CNN classifier trained on raw data provided the best performance with 2 convolution blocks after 100 epochs of training. Meanwhile, the classifier trained with generated samples had its best performance with 3 convolution blocks and 300 training epochs. In addition to that, same subject evaluation and cross-subject evaluation demonstrated the improvements attained by the classifier of augmented data in real test data samples with 3.28% and 5.18% AUC enhancement, respectively.

Another study by members of the same group continued using GANs to synthesize EEG data from the RSVP experiment [45]. They used WGAN-GP, and instead of cWGAN, the network was extended to a class-conditioned variant that performed event classification along with sample generation (Cc-WGAN-GP). The implementation of the proposed model was performed by employing different toolboxes Python 3.6.4, Tensorflow 1.12, and Keras 2.2.4. In addition, they proposed a log-likelihood score based on Gaussian mixture models as a quality evaluation of generated samples. They showed that the interpolation method for upsampling decreased the signal amplitude, meanwhile, it was more acceptable than deconvolution in sustaining the signal frequency. Thus, employing a two-step upsampling method, with bicubic interpolation followed by deconvolution with bilinear weight initialization, successfully enhanced GAN training. Nevertheless, it is important to mention that lop-sided amplitude appeared in the generated samples which are asymmetrical amplitude on edges. This issue was firstly handled in the upsampling layer by generating higher-dimensional EEG samples, then cropping it in subsequent layers to restore the generated samples with the same real sample dimension. Although the Cc-WGAN-GP classifier was deeper and more complicated than EEGNet, it had better performance mainly because of the GAN-based data augmentation. The CC-WGAN-GP average AUC was better than EEGNet for each subject, where the performance enhancement was between 0.28 and 16.4% with an average AUC of 82.98%, while EEGNet got only 77.16%.

Based on two prevalent GAN methods, namely DCGAN and WGAN-GP, Kunanbayev et al. proposed using data augmentation to generate artificial training data that were used in the classification of P300-EEG signals [42]. They used Arico et al. dataset [49] from a P300-based BCI Speller system with overt attention mode. For assessment of the generated data quality, the GAN-test / t-SNE visualization was performed. In fact, the real and generated samples from the same class were in the same group, and the different classes were totally separated. By implementing subject-specific augmentation, WGAN-GP resulted in a slightly higher performance compared to DCGAN and the classification improvement performance reached 11% for some subjects with an average enhancement between 2 and 4%. Ultimately, subject-independent augmentation, which followed the LOSO principle, achieved better classification performance with respect to the baseline classifiers (LDA and CNN) without augmentation for a relatively small size of the real training data (*n* = 50). On the contrary, for the bigger sample size (*n* = 288), baseline accuracy was better. Furthermore, WGAN-GP again had a better performance than DCGAN.

### Emotion recognition

Emotion recognition, an essential branch of emotion computing, plays a remarkable part in discovering people’s thoughts and understanding their behavior. Mainly, artificial emotional intelligence aims to develop tools, devices, and systems that enable the recognition of human emotions. This field of study gained noticeable attention as it builds this kind of bond between humans and machines. Emotion recognition based on EEG has proven its reliability and substantial accuracy compared to other approaches of emotion recognition that are based on facial expressions and gestures [50]. Recently, multiple studies have demonstrated the relationship between emotions and some mental diseases such as depression and autism [51, 52]. Consequently, these studies have considerable potential for treating psychiatric diseases. Emotion recognition techniques aim to identify two main parameters that define the underlying emotion: valence, which represents varying from unpleasant feelings to pleasant, and arousal, which is feeling variation from calm to excited/activated [53]. For benchmark EEG emotional databases, there are a few numbers of datasets for emotional EEG such as The Database for Emotion Analysis using Physiological Signals (DEAP) [54], The SJTU Emotion EEG Dataset (SEED) [55], and MAHNOB-HCI [53]. In DEAP, participants evaluated music videos in terms of the levels of arousal, valence, like/dislike, dominance, and familiarity. In SEED, subjects were asked to complete a questionnaire about their emotional reactions to film clips. For MAHNOB, participants’ responses to movies, images, and videos with correct or incorrect tags associated with human actions were recorded. With this intention, some studies considered using GANs to overcome the data scarcity problem in EEG emotion recognition. The shortage of data could lead to difficulty in building an accurate model with accepted accuracy using machine learning algorithms or deep neural networks. Table 3 summarizes different studies that used GANs to achieve an improvement in the emotion recognition field.

**Table 3:** Reviewed papers that used GANs in emotion recognition tasks

Study	Purpose	Dataset	GAN type	Evaluation metrics	Results (with GAN)
Y. Luo et al., 2018 [56]	Enhance EEG-based emotion recognition	SEED + DEAP	cWGAN	Classification accuracy	SEED: + 2.97% DEAP-Arousal: + 9.15% DEAP-Valence: + 20.13%
Y. Luo et al., 2018 [57]	Enhance EEG-based emotion recognition for semi-supervised models	SEED + DEAP	WGANDA	Classification accuracy	(W.R.T: SVM) SEED: + 30.43% DEAP-Arousal: + 17.63% DEAP-Valence: + 17.63%
Y. Luo et al., 2020 [59]	Enhance EEG-based emotion recognition	SEED + DEAP	cWGAN + sWGAN	Classification accuracy	SEED cWGAN + DNN: + 8.3% sWGAN + DNN: + 10.2% DEAP cWGAN + SVM: + 3.5% sWGAN + SVM: + 5.4%
Dong and Ren, 2020 [60]	Enhance EEG-based emotion recognition	DEAP	MCLFS-GAN	Classification accuracy	(w.r.t CNN + LSTM) SAP MCLFS-GAN: + 14.95% LOSO MCLFS-GAN: + 19.52%
Fu et al., 2021 [62]	Achieve a fine mapping of EEG data directly to facial images	SEED	Ac-GAN	• Classification accuracy • Reliability • Entropy	• Generated images from EEG 82.14% • 92.02% • 7.41
Liang et al. 2021 [63]	Fuse the spatial and temporal dynamic brain information into a better feature representation	SEED + DEAP + MAHNOB-HCI	CNN + RNN + GAN	• Classification accuracy • F1 score	• Up to + 7.69% • Up to + 5.07
Pan and Zheng 2021 [64]	Enhance EEG-based emotion recognition with sample scarcity and category imbalance issues	DEAP + MAHNOB-HCI	PSD-GAN	Recognition accuracy	• 2-classification task: 5.25%: 6.71% • 4-classification task: 10.92%: 14.47%
Chang and Jun 2019 [65]	Recognize the emotional responses of users towards given architectural design	Chang, Dong, and Jun Dataset [66]	GAN	Classification accuracy	+ 0.5%

Luo et al. proposed using the Conditional WGAN (cWGAN) framework for EEG-based emotion recognition DA [56]. A gradient-penalty version of WGAN is implemented to generate artificial EEG differential entropy (DE) features from noise distribution. They used three evaluation metrics to assess the quality of the generated data (Discriminator loss—Maximum Mean Discrepancy (MMD) – 2-D mapping). The results showed the rapid convergence of the discriminator loss to a small value (close to 0) for each subject. Moreover, the emotion recognition frameworks that were trained using the cWGAN generated and raw data, in comparison to using the raw data only, achieved improvements of 2.97% on the SEED dataset, and an improvement of 9.15% and 20.13% on the DEAP dataset for both arousal and valence classifications, respectively.

Another study by the same group proposed a modified framework of a WGAN domain adaptation (WGANDA) [57]. Their framework intended to recognize the new subject’s emotions with unlabeled data and reduce the gap between the probability distribution of different subjects caused by inter-subject differences that limit the generalization of trained models [58]. This framework mainly consisted of GAN-like components and a two-step training procedure with pre-training and adversarial training. The pre-training was used to map the source domain (labeled data) and the target domain (unlabeled data) to a common feature space, and the adversarial training was used to reduce the gap between the mappings of the source and target domains on the common feature space. For achieving more stability and rapid convergence of the framework, a WGAN-GP loss was adopted for adversarial training. Different accuracy comparisons were conducted to evaluate the proposed framework. First, the accuracy comparison between using adversarial-training (WGANDA-Adv.) and without adversarial-training (WGANDA-Bas.) was performed on the SEED dataset. Another comparison using three domain adaptation methods namely kernel principal component analysis (KPCA), transfer component analysis (TCA), and transductive parameter transfer (TPT) was conducted. Finally, the source and target data from SEED at different training stages were visualized in a 2D representation by the visualization tool t-SNE. The results demonstrated that the discriminator loss converges to a small value during training and WGANDA outperformed the state-of-the-art domain adaptation methods in terms of stability in the convergence process. Finally, the study illustrated the ability of the framework to recognize the emotions of a new subject with unlabeled data more precisely. Furthermore, they attained an improvement of (with respect to SVM) 30.43%, 17.63%, and 17.63% on SEED, DEAP arousal, and valence, respectively.

A more recent study by Luo et al. proposed and compared three EEG DA methods for emotion recognition: conditional WGAN (cWGAN), selective VAE (sVAE), and selective WGAN (sWGAN) [59]. The generation of realistic-like EEG training data had two forms: PSD and DE. The augmentation of the original training datasets was performed with different portions of generated EEG data, either full or partial usages of the generated data. A comparison was conducted with cVAE, Gaussian noise (Gau), and rotational DA (RDA) methods. Whilst the cVAE has a similar generated strategy as cWGAN, Gau depends on adding Gaussian noise to the original data for data augmentation. The RDA generates data from a geometric rotation of the original data. The results asserted the outperformance of generative models with respect to the aforementioned DA strategies. sWGAN achieved its best mean accuracy of 90.8% for SEED with DE features when 10,000 samples were added, while cWGAN achieved its highest mean accuracy of 87.4% when 15,000 samples were added. However, the cWGAN convergence speed was the quickest. For the DEEP dataset with DE features, cWGAN had its highest mean accuracy of 48.9% when 5000 samples were appended, whereas sWGAN achieved a higher mean accuracy of 50.8% with 15,000 appended samples.

Along with that, Dong et al. introduced a new design for emotion recognition named multi-reservoirs feature coding continuous label fusion semi-supervised GAN (MCLFS-GAN) [60]. They used a method to preserve the dependence of time sequence per every time window where the sample size is divided into windows of fixed length. Then, a sliding window technique was applied where the sliding of time windows was performed according to a certain sliding step set in the experiment. The sliding step is supposed to traverse the whole-time window size. The features were partitioned based on the spatial distribution and frequency band of EEG channels and then they were used to train by the multi-reservoir encoder. The multi-reservoir structure was used, based on a type of RNN named Echo-state networks [61], to decrease the interference of irrelevant feature data and the loss of critical feature information on the basis of keeping time characteristics in the window. Also, the spatial, temporal, and frequency characteristics of EEG signals were combined by the semi-supervised learning framework. In addition, the transfer learning concept was adopted to learn the mutual feature space representation of different subjects. Thus, the spatial representation, between the source domain and the target domain, was domain invariant. Meanwhile, continuous label fusion was done with respect to the degree of intensity of emotional category tags in the inner class. Therefore, reliable sample information was learned and increased the stability. The DEAP dataset was used in this research under two schemes. The first scheme involved sample shuffling, followed by tenfold cross-validation (tenfold CV) and LOSO as the second scheme. The results of the proposed method were compared to other methods such as LSTM + CNN, CNN + LSTM, L1-norm + SVM, SAE + LSTM, graph-regulated extreme learning machine (GELM), DANN, and ACGAN. Also, the classification accuracy of the proposed MCLFS-GAN is 81.32% and 54.87% with an overall enhancement reached of 14.95% and 19.52% (w.r.t CNN + LSTM) by using SAP and LOSO in the DEAP database, respectively.

Another cGAN method was proposed by Fu et al. [62]. They interpreted the EEG emotional data into a fine-grained facial expression image. In the proposed method (Ac-GAN), instead of quantitative evaluation, fine-grained facial expressions were assessed qualitatively from EEG signals. Mainly, the study followed a coarse-to-fine training strategy for Ac-GAN. In other words, they assigned five facial expression images (from the lowest level of emotion to the highest level) as the target images with the same coarse label, then Ac-GAN was trained on the EEG data and learned its distribution characteristics. By ranking coarse EEG data samples from strong to weak associated with the measured beta and gamma intensities, it learned fine-grained information and generated the fine-grained facial expression image. Results demonstrated the reliability of the generated positive and negative facial expression images with a classification accuracy of 93.77% and 90.26%, respectively, and the classification accuracy on four classification tasks (low/ high positive and low/ high negative) was significant with 82.14%.

Liang et al. revealed the effectiveness of GAN-based methods as an unsupervised fusion model with a reliable across-subject emotion recognition performance [63]. They proposed a novel unsupervised EEG feature extraction method (EEGFuseNet). EEGFuseNet architecture consists of a hybrid network of CNN, RNN, and GAN. CNN extracts features from raw EEG signals, and RNN detects the feature relationships at every time point which improved the feature representation by combining both temporal and spatial information. Then, GAN was used to enhance the training process of the CNN-RNN network with dynamic updates in an unsupervised manner, for generating high-quality features. The generator was a CNN-based encoder-decoder network and the CNN-based discriminator was used to distinguish between generated and real samples. They also adopted a LOSO cross-validation subject-independent protocol. Concerning the role of GANs, the comparison between the CNN model only, the CNN + GAN model, and the CNN + RNN model only elucidated the impact of GAN on improving the accuracy performance and F1 score. Using CNN-GAN on the DEAP database increased the accuracy between 0.4 and 2.96% for dominance and the F1 score by 3.47 to a maximum improvement of 4.26 for dominance. In addition, EEGFuseNet outperformed the CNN-RNN network (without GAN) by 1.1% to 1.93% for accuracy and 1.77 to 2.05 for the F1 score. Finally, the results of the SEED and MAHNOB-HCI datasets supported the DEAP dataset results as the accuracy increased by 7.69% for Arousal of MAHNOB-HCI and the F1 score gained an increase of 5.07 for the SEED dataset.

In [61], Pan and Zheng proposed PSD-GAN using a generator that comprises three linear layers using two ReLU functions and one Tanh function and the discriminator with two linear layers with a LeakeReLU function and a sigmoid function. PSD here indicates that GAN generates samples with PSD features. Using the DEAP dataset for evaluation, the accuracy for a single subject was improved by 5.25% and 6.38% on average in two-classification tasks (high–low) and by 6.5% and 6.71% on average across subjects in valence and arousal recognition, respectively. Similarly, in the four-classification task (High/ Low Valence and High/ Low Arousal), the accuracy improvement was 10.92% for a single subject and 14.47% across subjects. In addition, the study investigated the emotion recognition performance by exploiting MAHNOB-HCI dataset with two feature extraction models frequency band correlation (FBCCNN) and frequency band separation (FBSCNN) with and without synthetic data generation. It was noted that the FBSCNN accuracy was enhanced from 56.78% by PSD-GAN to 66.50%. Furthermore, with FBCCNN the accuracy increased from 62.06 to 70.34%. Overall, the proposed model improved learning features from several categories and subsequently reduced overfitting, increased generalization capability, and attained a better recognition rate. Finally, the framework was implemented by PyTorch and on the Nvidia Titan RTX GPU in a fully supervised manner.

For the assessment of user feedback toward visual designs, architects applied GAN techniques with EEG signals to explore the user’s feedback toward their designs in [65]. The proposed frameworks consisted of two main algorithms including GAN for EEG data augmentation, and a deep neural network model to classify the emotional states of the EEG signals as "positive" or "negative" toward the design. The study used the dataset from [66] of 18 subjects for evaluating the proposed framework. The implementation of the GANs model was carried out by TensorFlow platform utilization. The results revealed that fusing such a technique was promising as the final accuracy increased by 0.5% to reach 98.4% by using the generated artificial data with raw data. The study opened up a new avenue of using EEG signals to help the designer in the architectural design step and to detect the emotional responses of clients towards suggested design alternatives.

### Epilepsy studies

Epilepsy is a chronic neurological disorder in which patients suffer from several seizures [67]. Due to epileptic seizures, patients experience several symptoms such as uncontrolled jerky movements, body convulsions, loss of awareness, and sensory auras [68]. As a result, epilepsy impedes the quality of a patient’s life and increases the mortality rate of patients with frequent seizures [69]. In addition, medications are not always the effective solution for a lot of epilepsy patients [70]. Hence, seizure detection and monitoring have a prominent role in patient diagnosis, improving the standard of living, and understanding of seizures. On the other hand, if those patients could be alerted before the occurrence of such seizures, it will give them the chance to take the appropriate precautions or control seizures with medications. In turn, epileptic seizure prediction is more likely to mitigate the seizures implication on patients and improve their quality of life.

Epileptic seizures could be classified into four main states. The normal state of brain activity is the interictal state, while the preictal state begins 60 to 90 min before the occurrence of seizures. Then, the ictal state starts with the onset and ends with the seizure, and finally, the postictal state starts after the seizure ends [71]. On that understanding, automatic seizure detection can be represented as a binary classification task that discriminates between EEG patterns of the ictal and non-ictal states, whereas epileptic seizure prediction can be represented as a binary classification task that discriminates between EEG patterns of the preictal and the interictal states.

Although machine-learning-based algorithms have offered a promising solution for both automatic seizure detection and prediction techniques, these algorithms often require a large number of training data points. Unfortunately, obtaining EEG signals during epileptic seizures is a process that could be considered very costly and time-consuming for both medical specialists and patients. Thus, creating synthetic seizure-like EEG signals is a suggested solution that could be used to train seizure detection and prediction algorithms. Among different generative data methods, GAN was introduced as a superior method for generating artificial data on epilepsy seizures to train seizure algorithms. Table 4 summarizes here the studies that used GAN-based algorithms for epileptic seizure detection and prediction.

**Table 4:** Reviewed papers that used GANs in epilepsy studies

Study	Purpose	Dataset	GAN type	Evaluation metrics	Results (with GAN)
Wei et al., 2019 [67]	Proposes an automatic epileptic EEG detection method	CHB-MIT Scalp	WGANs	• Classification accuracy • Sensitivity • Specificity	• + 2.51% • + 1.43% • + 3.59%
You et al., 2020 [68]	Solve the class imbalance problem of epileptic seizures detection	Private dataset	DCGAN an anomaly detector	• AUROC • Sensitivity • False detection rate	• 93.93 1% (With Gram Matrix) • 96.3% • 0.14 per hour
Pascual et al., 2021 [70]	Overcome scarcity of epileptic seizures EEG signals and address the privacy concerns	EPILEPSIAE project [73]	Epilepsy GAN	• Classification accuracy • Synthetic data Recall values • Geometric mean of sensitivity and specificity	• + 1.3% • median: + 3.2% • + 1.3%
Truong et al., 2019 [74]	Predict seizures with an unsupervised algorithm	CHB-MIT + Freiburg Hospital + EPILEPSIAE	DCGAN feature extractor	• Classification accuracy	CHB-MIT: 61.53% Freiburg Hospital: 53.84% EPILEPSIAE 13.33% (with AUC above 80%)
Usman et al. 2021 [71]	Solve the class imbalance problem of epileptic seizures predictor	CHBMIT	GAN	• Sensitivity • Specificity • Anticipation time	• 93% • 92.5% • Average 32 min
Usman et al., 2021 [75]	Overcome the challenge of accurate prediction of epileptic seizures	CHBMIT + American epilepsy society-Kaggle seizure prediction	GAN	Classification accuracy	• CHB-MIT: + 1.74% • IEEG: achieved 95.53%
				Sensitivity	• CHB-MIT: + 1.56% • IEEG: 94.27%
				Specificity	• CHB-MIT: + 1.93% • IEEG: 95.81%
				Average Anticipation Time	• CHB-MIT: 1.34 m
Salazar et al., 2021 [76]	Improve seizure prediction performance with extreme data scarcity	Private dataset “Barcelona test”	GAN + vector Markov Random Field (vMRF)	Classification accuracy	NA
Rasheed et al. 2021 [77]	Improve seizure prediction performance	Epilepsyecosystem + CHB-MIT	DCGAN	• Sensitivity • AUC	• + 15% • + 6:10%

In [67], automatic epileptic EEG detection using WGANs was introduced. The authors proposed a method based on CNN that automatically extracts features of raw data after learning the representation. They used the merger of the increasing and decreasing sequences (MIDS), a time-domain merging method for signal processing that highlights waveform features with respect to human vision measurement [72], to process the EEG signals for the training of CNN in comparison with the baseline CNN model. WGAN is then used to add sample diversity and generate more EEG data which could overcome the class imbalance problem, especially in case of increasing negative sample data. To evaluate the approach, the LOSO approach was adopted. Using the CNN with MIDS improved the accuracy, sensitivity, and specificity in comparison to the use of CNN applied to raw EEG by 1.78%, 3.4%, and 0.16%, respectively. On the other hand, using the CNN with DA attained the best accuracy of 84% with a sensitivity of 72.11% and a specificity of 95.89% which was higher than what was achieved using CNN with raw EEG by 2.51%, 1.43%, and 3.59%, respectively.

For unsupervised learning, another approach for automatic seizure detection was introduced by You et al. [68] that uses the DCGAN technique. DCGAN training was performed using unsupervised learning and the evaluation followed the behind-the-ear EEG recording method. They recorded behind-the-ear EEG by two pairs of electrodes from 12 patients with various types of epilepsy. The recorded EEG signals were utilized to create a PSD image for the two channels of two electrode pairs (channel 1 (left–right temporal), channel 2 (left–right central)), and the third additional virtual channel (channel 3) of the two channels’ means. Then, the recorded dataset was examined to distinguish the onsets and ends of seizures. The study first used GAN unsupervised learning of the normal records to know the representation of normal states. Subsequently, to solve the imbalance problems, they adopted automatic seizure detection with the trained DCGAN as an anomaly detector to identify the ictal events in epilepsy patients. Finally, they used the combination of the Gram matrix with other anomaly losses to improve detection performance. By conducting unpaired t-tests for the anomaly’s comparison between normal and ictal samples in the dataset, both channels 1 and 3 illustrated significant differences for all EEG bands. Meanwhile, the delta, theta, and alpha bands of channel 2 showed significant differences. The area under the receiver operating curve reached 0.939 with a sensitivity of 96.3% and a false alarm rate of 0.14 per hour in the test dataset. The whole algorithm was implemented using Python 3.5 with TensorFlow.

Pascual et al. proposed a GAN model to generate synthetic ictal data for seizure detection [70]. They aimed to tackle privacy issues related to patient medical data in epileptic seizures. They adopted a strategy known as “Train on Synthetic, Test on Real '' to assess synthetic ictal samples. Further, the evaluation employed an advanced classifier with an RF algorithm by using the EPILEPSIAE project database [73]. The generator was a U-net convolutional autoencoder network with weighted skip connections. In general, the autoencoder has two symmetric parts, the encoder where the input samples are processed and a latent code is produced, and the decoder which decodes the latent code and gets the original sample. In this study, the decoder is employed to interpret the latent code into an ictal sample. Of equal importance, the synthetic samples training achieved an accuracy higher by 1.3% compared to RF training with real samples only. Using the synthetic ictal signals achieved a mean accuracy of 95.4%. For privacy concerns, when synthetic ictal signals were different across patients, identifying the patient from synthetic ictal signals was more difficult than from real ones, without affecting the seizure detection task. Particularly, synthetic data was around 7.2 times less vulnerable to re-identification compared to real data.

For seizure prediction, Truong et al. proposed an approach for predicting seizures using unlabeled EEG signals [74]. This study utilized the short-time Fourier transform on 28 s EEG windows as a pre-processing step. GAN was used in a different way as the DCGAN’s discriminator is used to extract features from unlabeled EEG signals. The extracted features are then classified by a neural network classifier consisting of two fully connected layers for the labeled EEG signals and this classifier could be replaced by any other classifier. Moreover, Seizure Occurrence Period (SOP) and Seizure Prediction Horizon (SPH) were used. SOP is the time interval when the seizure is more likely to happen, and the interval between the time point at which a seizure is predicted and the SOP is called SPH. These two measures were adopted as follows: SOP of 30 min and SPH of 5 min for evaluation. In addition, a comparison of GAN methods in three different scenarios with CNN was held: trained GAN with data of all patients (GAN-NN), trained GAN in a patient-specific (GAN-PS-NN), and with upsampling (GAN-PS-USPL-NN). Meanwhile, it is worth mentioning that although the feature extraction of seizure prediction was employed in an unsupervised way, the overall area under the operating characteristic curve (AUC) decreased by ∼ 6–12% across the three datasets with respect to fully supervised CNN. Nevertheless, it was noted that the average AUC increased to 75.66% and 74.33% for the CHB-MIT dataset and the Freiburg Hospital dataset, respectively, by utilizing 10 times the size of dataset upsampling, which are 1–2% lower than those of supervised GAN-NN. Therefore, training GAN with the upsampled inputs alleviated this difference between fully- and semi-supervised paradigms for several patients and increased the performance. The model training is performed on an NVIDIA P100 graphic card using Tensorflow 1.4.0 framework. Finally, the researchers showed that both supervised and semi-supervised learning methods (CNN, and GAN-PS-USPL-NN) were better than the random predictor for almost all patients as the prediction performance for the two methods reached (92.30%, 84.61%), (100%, 84.61%), and (86.67%, 86.67%) for the three datasets. For the semi-supervised patient-specific method, it had an AUC of 77.68%, 75.47%, and 65.05% for the CHB-MIT scalp EEG dataset, the Freiburg Hospital intracranial EEG dataset, and the EPILEPSIAE dataset, respectively.

Recently, Usman et al. suggested two GAN-based methods for seizure prediction [71, 75]. First, they designed a GAN to generate preictal samples with empirical model decomposition and three CNN layers of automated feature extraction [71]. In this study, an LSTM classifier was utilized for the classification of interictal and preictal states and they used the CHBMIT dataset of scalp EEG signals for evaluation. The results of this method yielded 93% sensitivity and 92.5% specificity with an average time of 32 min to predict the seizure's onset. Furthermore, the proposed GAN was successfully able to resolve the class imbalance problem with data having a similar distribution to the original one.

In [75], the authors proposed another method for extracting both handcrafted and automated features by a modified three-layer CNN that follows signal preprocessing. Feature selection is performed to get a comprehensive feature vector. Furthermore, the Model Agnostic Meta-Learning (MAML) classifier was proposed to reduce the number of training examples for the classifier by receiving output from three different classifiers SVM, CNN, and LSTM which resulted in increasing classification accuracy without overfitting. Two datasets of scalp EEG and intracranial EEG were used (CHBMIT, American epilepsy society, and Kaggle Seizure Prediction Challenge intracranial EEG dataset) for evaluation of this method. The results were remarkable as the average sensitivity reached 96.28% and the specificity reached 95.65% with an average anticipation time of 33 min on all subjects of CHBMIT. Moreover, the proposed method improved the accuracy from 74% with handcrafted features and SVM to 96.05% using EMD, bandpass filter, GAN, handcrafted and CNN-based features, feature selection using PCC and PSO, and an ensemble classifier. Along with that, the American epilepsy society-Kaggle seizure prediction dataset resulted in an average sensitivity of 94.2% and specificity of 95.8% with a mean accuracy of 95.53%.

Combining two basic concepts: GAN and vector Markov Random Field (vMRF), Salazar et al. proposed another method (GANSO) for oversampling the classifier training set [76]. The main concept of vMRF, which is just an MRF extension, depends on linking samples of the data that were presumed to be correlated. It acts as a type of regularization to enhance the synthetic samples generated from the original ones. Meanwhile, vMRF was used by GAN to generate samples with the Graph Fourier Transform (surrogating approach). Thus, to oversample the classes' instance space, different classes shared the same vMRF. Then, the discriminator block was a linear classifier to obtain features similarities between the generated and the original samples. Different evaluation criteria were adopted for the introduced method such as the classification of the neuropsychological activity test (Barcelona test) using EEG data from epileptic patients, in addition to other physiological data. The GANSO findings were remarkable as they decreased the probability of error for most random detectors with a very little training set size (only 3 or 5 original instances). In addition, the learning curves of error probability decline were rapid with the added number of generated signals equal to two or three times the available number of original signals. On the other hand, Synthetic Minority Oversampling Technique (SMOTE) was not able to have an acceptable result with such a small size training set.

Using a different approach, Rasheed et al. proposed a DCGAN model for generating both artificial scalp EEG data and intracranial EEG (iEEG) data for epilepsy seizure prediction [77]. They used the CHB-MIT dataset and the Epilepsyecosystem dataset to train the DCGAN and evaluated the algorithm accuracy for SPH of 10 min and SOP of 30 min. Then, SVM and designed convolutional epileptic seizure predictor (CESP) classifiers were utilized to evaluate the proposed model. They trained a one-class SVM on real data, then used it for testing the generated samples and picked those real-like synthesized samples. Furthermore, by training the CESP model on the augmented dataset (5 and 3 times the dataset size), the comparison with unaugmented data depicted that DA by DCGAN increased the sensitivity almost by 15% and AUC by 10% and 6% for Epilepsyecosystem and CHB-MIT datasets, respectively. In addition, the DCGAN with CESP classifier model was compared to other epilepsy prediction models that used traditional augmentation techniques like SMOTE, moving windows, and data sampling. The proposed model had the best sensitivity of 96% and 92.87% for both datasets and it was implemented by utilizing Keras toolboxes.

### Other EEG applications

The previous sections have not covered all studies that have tried adopting GAN methods for EEG signals. Thus, this section includes a wider scope of using GAN with EEG data. Researchers have proposed GAN in studies including Steady State Visual Evoked Potential (SSVEP) tasks, protecting EEG data from illegal access, augmentation of various types of Biosignals, classification of sleep states, and classification of fatigue during driving. Besides, short, and long-time series predictions of EEG signals have been addressed in addition to imputing missing signal sequences. Table 5 summarizes all these studies.

**Table 5:** Reviewed papers that used GAN in other EEG applications

Study	Purpose	Dataset	GAN type	Evaluation metrics	Results (with GAN)				
Khadijah et al. 2019 [18]	Improve the accuracy, convergence rate, and generalization capabilities of the model	Private dataset + Nao dataset	DCGAN + WGAN	Classification accuracy	G + R data train DCGAN: + 3% WGAN: + 2%	G data train DCGAN: + 1% WGAN: + 3%			G data train generalization DCGAN: + 12% WGAN: + 11%
Yao et al., 2020 [80]	Specify several standards for operating on EEG data to protect users’ privacy	From UCI, Neuro-dynamics Laboratory at the State University of New York	ResNet generator + patchGAN feature filter	Privacy indicator	Filter out over 90% of alcoholism information on average from EEG signals, with an average of only 4.2% useful feature accuracy lost				
Hazra and Byun, 2020 [82]	Eliminate confidentiality concerns of medical data	Siena Scalp + private dataset	SynSigGAN	• Pearson Coefficient • MAE • RMSE • PRD • FD	• 0.997 • 0.0475 • 0.0314 • 5.985 • 0.982				
Fan et al., 2020 [83]	Address the challenges of automatic sleep staging models such as the inherent class imbalance problem	Montreal Archive of Sleep Studies (MASS) + Sleep-EDF-SC	DCGAN	• Classification accuracy • F1 score • Cohen Kappa	MASS • 3.79% • 3.48% • 5.43%			Sleep-EDF • 4.51% • 3.14% • 5.8%	
Zeng et al., 2021 [86]	Address the issue of the different distribution of EEG across subjects	Private dataset	GDANN	• Classification accuracy • Precision • F1Score • Recall	• + 11.9% • + 9.34% • + 9.64% • + 10%				
Hazra et al., 2021 [87]	Develop a cost-effective system for cognitive state classification using ambulatory EEG signals	Private dataset	DCGAN Classifier	Classification accuracy	Compared to CNN • GTCC – MFCC: + 0.6% • GTCC – MFCC – CNN: + 0.3% • GTCC: – 1.33%				
Yin et al., 2021 [78]	Use multivariate time series data in the process of prediction	NASDAQ100 + SML2010 + Energy + EEG + Air Quality	MAGAN	• MAE • SMAPE	ɛ = 50 w.r.t MARNN • − 0.0455 • − 0.0244				
Yin et al.., 2021 [79]	Improve the accuracy of the long-term prediction	NASDAQ + SML + Energy + EEG + KDDCUP	VAEcGAN	• MAE • RMSE	ɛ = 120 w.r.t LSTM • − 0.1529 • − 0.1334				
Tazrin et al. 2021 [88]	Solve computational and energy resource issues of IoT devices with EEG headbands/headsets	Confused student dataset	DCGAN	Classification accuracy	CNN & DNN > + 20%				
Cheon et al. 2021 [90]	Overcome issues of gathering a large dataset of EEG	Confused student dataset	CTGAN + TGAN	• Basic statistics • Correlation column correlations • Mean correlation • 1 – MAPE • Similarity score	CTGAN • 0.9963 • 0.9476 • 0.9393 • 0.7250 • 0.9021		TGAN • 0.9876 • 0.0881 • 0.9351 • 0.8552 • 0.7165		
Lee et al. 2021 [91]	Improve classification of sleep stages	Publicly Sleep-EDF database (PSG) test	Sig-GAN	• Classification accuracy • IS • FID	• 65.67% with only first 30-s signals (Real data 82.85%) • − 0.51 (w.r.t real data) • + 39.53				

In 2019, Khadijah et al. used DCGAN and WGAN to generate augmented EEG signal vectors for training an SSVEP classifier with a variety of unsupervised models [18]. SSVEP is an evoked potential produced when a subject focuses on regularly flickering objects that flicker at specific frequencies [18]. It was found that the flickering frequency can be extracted from the subjects’ recorded EEG signals during flashing via various signal processing techniques. The proposed method generated EEG data directly in signal space via end-to-end training without transforming the signals into different domains. They employed two empirical SSVEP dry-EEG datasets for evaluation. It is worth mentioning that DCGAN, WGAN, and VAE models successfully captured the characteristic of SSVEP peaks at the target frequency with its harmonics. In the meantime, the generated signals from VAE had relatively smaller amplitude. In addition, using the generated and real data for training the classifier, DCGAN and WGAN achieved 3% and 2% classification accuracy improvement with respect to the baseline CNN model without synthetic data, respectively. In addition, the classification accuracy was improved, compared to the baseline model trained with raw data, by 1% and 3% for DCGAN and WGAN, respectively. Finally, all generative models were implemented by (PyTorch).

Yin et al. have considerable contributions in [78] and [79]. First, a multi-attention generative adversarial network (MAGAN) was proposed for multivariate time series prediction. This model had three main parts: the encoder, the generator or decoder, and the discriminator networks. The encoder network had an input-attention network for correlation extraction between target data and self-attention. Then, the long-term temporal relevance of hidden data was selected by temporal-convolution-attention through the decoder stage. Finally, convolution layers, based on the weight clipping algorithm, extracted data features and classified the generated data that had the true data. In addition, they used the encoder network and the decoder network as another method called multi-Attention based RNN (MARNN). Furthermore, experimental evaluations were performed on five real datasets including one EEG dataset. The recorded EEG dataset was data from subjects performing an SSVEP experiment. The proposed MAGAN was compared with different methods including LSTM, Seq2Seq, Temporal-att-RNN, DARNN, TCN, and MARNN. The comparison used the following measures: MSE, RMSE, MAE, mean absolute percentage error (MAPE), symmetric mean absolute percentage error (SMAPE) and ${R}^{2}$ score. The results of the proposed methods with the EEG dataset on the short-term prediction (predicted time steps = 1) showed the efficiency of the MARNN, especially with MSE, RMSE, and R-squared value Meanwhile, MAGAN showed its superiority in MAE with 0.2069 and 0.6635 of SMAPE. In addition to that, both the LSTM and seq2seq models kept temporal dependence, and the seq2seq model could successfully output indefinite length values. Overall, MARNN and MAGAN models had better performance for all datasets compared with the other aforementioned methods in short-term and long-term prediction, respectively.

Another study for long-term prediction was presented by Yin et al. in [79]. This study proposed VAEcGAN which consisted of the same three stages of the previous study: the encoder, the generator, and the discriminator. However, in this model, the encoder was a VAE one. Consequently, the latent space was not a random noise, instead, it had a part of the data of the driving series from the VAE encoded data. The generation stage exploited both LSTM and attention to generate prediction data with the equivalent time trend as the data from previous time steps. The discrimination stage had the same concept as convolution layers as it extracted data features and distinguished between the generated and true data. They followed the same evaluation methods used in [78], but this study differs only in terms of evaluation indexes, which were just MAE and RMSE, and the compared methods were: LSTM, Seq2Seq, DARNN, TCN, Dual-Stage Two-Phase based RNN (DSTPRNN), and VAE. It was shown that the DSTPRNN model performance was better than the VAE model without the cGAN module. However, the prediction results of the VAEcGAN model were clearly improved. For EEG dataset results, the VAEcGAN algorithm was more stable and accurate compared to other models in long-term prediction (prediction steps = 120) with the significantly least values of MAE and RMSE.

With an aim to protect EEG data from illegal hacking, another study was introduced to address feature fusion problems that occur with EEG signals using GANs [80]. It depends on mapping EEG signals with undesirable features directly toward EEG signals without those features which attain users’ privacy. Yao et al. proposed an end-to-end algorithm in which an image-wise autoencoder, based on Fast Fourier Transform (FFT) and CNN, was employed. Instead of extracting features from raw EEG data, three EEG frequency bands were selected to create an RGB-color image and then the autoencoder extracted features from those images with both classification loss and reconstruction loss. In addition, a GAN-based technique with a structure including a combination of ResNet generator and PatchGAN discriminator from Pix2Pix [81] was employed to generate new EEG signals without the undesired features. For the evaluation of the framework, the alcoholism dataset was used from UCI. It was found that the model can conceal over 90% of alcoholism data from EEG signals, with an average of only 4.2% useful feature accuracy lost.

A similar approach for protecting patient data and information was proposed by Hazra and Byun named SynSigGAN to generate various artificial biomedical signals from a modest dataset of real signals [82]. The model depended on refining the signals by employing a combination of discrete wavelet transform (DWT), thresholding, and Inverse discrete wavelet transform (IDWT) through preprocessing phase. Then, a GAN architecture, which consisted of a bidirectional grid of long short-term memory (BiGridLSTM) as a generator network and a CNN for the discriminator network, was used to augment the biosignals. The study involved four kinds of biomedical signals (ECG, EEG, electromyography (EMG), and photoplethysmography (PPG)). The Pearson correlation coefficient was utilized to evaluate the quality of generated data in addition to the following metrics for statistical analysis: the Root Mean Square Error (RMSE), Percent Root Mean Square Difference (PRD), MAE, and Frechet Distance (FD). The results for the EEG dataset demonstrated that the BiGridLSTM combined with CNN in the GAN architecture had the best results with 0.997, 0.0314, 5.985, 0.0475, and 0.982 for the aforementioned metrics, respectively.

Another study by Fan et al. compared five different DA techniques for sleep EEG signals [83]. Mainly, sleep consists of three main stages, including wake (W), rapid eye movement (REM), and non-rapid eye movement (NREM) stage that includes N1, N2, and N3 [84]. The classification of sleep stages could have a great effect on people’s lives to enhance and monitor their sleep. Actually, it reflects the mental and physical health of people [85]. While automatic sleep staging models require a large amount of data, this paper compared DA techniques as a potential solution including repeating minority classes (DAR), morphological change (DAMC), signal segmentation and recombination (DASR), dataset-to-dataset transfer (DAT), and DCGAN. In addition, they adopted a typical CNN architecture classification model to assess the performance of the aforementioned DA approaches with a sleep staging model on two datasets (the Montreal archived of sleep studies (MASS) / Sleep-EDF). Compared to the trained CNN classifier without DA, using GANs in particular successfully enhanced the overall classification performance as measured by using the accuracy, F1 score, and Cohen Kappa coefficient range (K) as evaluation metrics. Visual inspection and distance indicators also showed that the generated signals were real-like. Also, DCGAN performance outperformed other techniques, in most cases, as it achieved 0.767, 0.692, and 0.656 on the MASS dataset and 0.748, 0.685, and 0.660 on the Sleep-EDF dataset for ACC, F1 score, and K, respectively. However, the study posited that DCGAN methods suffered from the complexity and cost of resources for training compared to other DA methods. The models were implemented using Pytorch.

Zeng et al. introduced a new transfer learning model of Generative Domain Adversarial Neural Network (GDANN) for detecting fatigue during driving [86]. This model utilized GAN to improve the EEG analysis with various subjects’ distributions. The architecture of this model consists of a combination of the DANN, including its three networks (Feature Extractor—Label Predictor—Domain Classifier), and GAN. First, they modified the optimizer and the loss function of DANN in the hidden layer to map data from various distributions of different sources to the target domain. Then, GAN used random noise to generate fake data similar to the data distribution in the target domain resulting in the enhancement of the model training by balancing the dataset in the source and target domains. The feeding with different source domain data enables GDANN to choose the samples that had the best similarities for target data distribution. A comparison between the proposed model and other models such as DANN, SVM, and Easy Transfer Learning (EasyTL) was held. Cross-subject cross-validation process revealed that GDANN had the best average accuracy of 91.63% in fatigue detection across subjects. In addition, the GDANN effect was obvious with respect to the original model DANN as it attained significantly higher classification accuracy by 11.9%. Nonetheless, the proposed model was noted as more time-consuming compared to other methods where it was implemented by Python 3.6.8 tools under a Linux environment with Ubuntu 5.4 operating system.

Hazra et al. proposed three different feature extraction methods for EEG cognitive state classification, Gammatone Cepstrum Coefficients (GTCC), a combination of GTCC and Mel Frequency Cepstrum Coefficients (MFCC), and 1D CNN model to extract features after the ensemble (GTCC + MFCC) feature space [87]. Moreover, a 1D DCGAN model was employed as a classifier based on the proposed methods GTCC, GTCC + MFCC, and GTCC + MFCC + CNN. With a novel proposal of EEG data collection protocol, external vision stimuli from multiple sources were used for evaluating the aforementioned methods such as EOS (Emotion Oriented State), MOS (Memory Oriented State), ROS (Relaxing Oriented State), TOS (Thinking Oriented State), SROS (Simple Regular Oriented State) and IOS (Illness Oriented State). The study included a comparison with other feature extraction methods such as Discrete Wavelet Transform (DWT), MFCC using Fisher Discriminant Ratio (FDR), and Logistic Regression (LR) statistical metrics. Furthermore, multiple classification models were used to obtain the performance accuracy on extracted features like Probabilistic neural network (P-NN), LDA, Multi-Class SVM (MCSVM), Decision Tree (DT), and RF. The results of this study demonstrated that the 1D DCGAN model classifier had a better performance than the basic CNN model. The proposed GTCC + MFCC achieved with the DCGAN model an accuracy of 96.42%, similar to the GTCC + MFCC + CNN performance with an accuracy of 96.14%. Meanwhile, both models were better than the GTCC alone with 87.79% accuracy. Furthermore, the accuracy of all proposed models with the base-CNN classifier was lower than using the DCGAN classifier.

Also, Tazrin et al. adopted DCGAN for increasing their dataset size with their proposed model called Logic-in-Headbands-based Edge Analytics (LiHEA) [88]. The DCGAN of this model was implemented by three 1D convolutional layers followed by three LeakyReLU layers for the generator and the discriminator where various IoT devices like Raspberry Pi 3, and Raspberry Pi 4. NVIDIA Jetson Nano has been utilized. In addition, the dataset of confused student EEG signals from a public repository [89] was employed to train the three algorithms: RF, DNN, and CNN. The dataset includes the student’s recorded EEG signal while watching educational videos as a mental health assessment and indicator for concentration levels. Although augmented data by DCGAN had a negative effect on the RF model, the performance of both DNN and CNN was considerably improved by about 20% using the artificially synthetic data of EEG samples. In fact, the generated data with DNN led to classification accuracy enhancement from 54.9 to nearly 80% when the training data increased by 4 times. Meanwhile, it was noted that the proposed DCGAN model increases the training complexity of the LiHEA model.

Cheon et al. presented their study of conditional tabular GAN (CTGAN) and tabular GAN (TGAN) for creating synthetic data [90]. TGAN is constructed of an LSTM as a generator and a feed forward NN discriminator. Meanwhile, conditional vector and generator loss were applied for solving imbalance problems in CTGAN. Again, the confused student EEG dataset [89] was used for the assessment of both methods. By adopting different visualization methods for evaluation such as the column-specific sum, distribution of real and synthetic data, and column-specific differences, the similarity between real and artificial data, the generated data was validated. Moreover, the similarity score, which is the average of correction column correction—mirror column correction—1-MAPE estimator—1-MAPE PCA, confirmed that CTGAN generated more real-like data than TGAN. Finally, the authors fed the generated data to RF, XGBoost, LightGBM, and Catboost algorithms. In turn, the RF classifier using the TGAN data attained the highest accuracy compared to all other algorithms. However, using both TGAN and CTGAN failed to achieve better accuracy compared to the algorithms without generated data.

Lee et al. proposed SIG-GAN for imputing missing signal sequences in EEG data [91]. The architecture of the Sig-GAN included the encoder of a generator which employed two sequences of convolutional layers in parallel and its input signals pass through two different 1-dimensional CNN. Furthermore, two layers of transpose CNNs for the decoder were designed with the discriminator and auxiliary network which were stacks of convolutional layers and consisted of a fully connected layer. They used the publicly available Sleep-EDF database [92] to evaluate their technique. By using the DeepSleepNet classifier [93], SIGGAN yielded 65.67% classification accuracy of sleep stage scores with only the first 30-s signals compared to the real data accuracy of 82.85% without imputation. In addition, the technical evaluation was conducted by comparison with two other methods of imputing missing data (with random sampling and another WGAN of [27] methods) by varying the ratio of missing data in the signal sequences (missing from 0 to 50%). Eventually, the results confirmed that SIGGAN remarkably outperformed the other methods with almost all ratios of missing data. On the other hand, the classification accuracy of the other imputation algorithms dramatically falls with removing data by 48%; SIGGAN achieved 75.75% and about 78% with DeepSleepNet and SleepEEGNet [94] classifiers, respectively. Meanwhile, the model got only 45.05% accuracy without an adversarial loss (GAN loss) which demonstrates the necessity for the GAN part of the model. Finally, all deep neural networks using have been implemented by TensorFlow 2 on Python 3.7.

## Discussion

This overview of the state-of-the-art GAN models for EEG elucidates the enviable contributions of GANs to solve the issues of scarcity and limitation of small-scale datasets in various EEG tasks. GANs have demonstrated success in augmenting EEG data for motor imagery, P300-based applications, emotion recognition, and epileptic seizure detection and prediction. It is noteworthy here that a few studies on EEG-based image generation have been excluded from this article such as [95–98], and [99, 100]. The main justification for this elimination is that these studies mainly use GAN methods for image generation and EEG signals were being used as an auxiliary input without applying GAN to the EEG data itself. Throughout this overview, it is noticeable that different versions of WGAN have demonstrated their superiority to generate EEG data and improve the performance of the aforementioned tasks.

Despite the efficacy of GANs, few considerations have been noted for using GANs with EEG signals and need to get more attention and investigations in future studies. First, the existence of high-frequency artifacts in the generated samples has not been sufficiently explored. These artifacts have an obvious implication for the model’s performance [101]. However, few studies through our reviews paid attention to these artifacts in generated signals and aimed to alleviate their impact. Apart from that, the quality metric for the generated EEG data is still a heated-up issue. To date, there is no direct metric that could be considered a real assessment of the generated EEG signals’ quality and directly relate it to the performance of the model [21, 27]. IS and FID were used as metrics for clarity and variety of the generated signals. In addition, WD and ED were also used as similarity indicators. Nonetheless, IS is sensitive to noise, and both IS, nor FID are not able to deal with overfitting [101]. Furthermore, WD increases complexity, and ED with several attributes could be imprecise [101]. Second, the number of artificially augmented data samples has a remarkable effect on the performance of the classifier after data augmentation [39]. It was noted that after a specific number of generated data samples, there is a drastic variation in the model performance either positively or negatively. Moreover, the number of available training set data points is believed to represent an issue for GAN performance in the case of extremely small datasets [102]. Third, the use of GAN for preserving patient privacy by data augmentation showed that identifying the patient from synthetic signals was more difficult than from real ones. However, there is always a trade-off between data quality and data privacy as concealing more features results in worse quality of data. Fourth, another challenge that is worth exploring is employing GANs in unsupervised approaches. Although GAN was successfully adopted in an unsupervised manner and attained accepted results in each task [18, 57, 63, 68, 74], further investigations of GAN for unsupervised algorithms are still required to enhance the performance and achieve these results that compete with the supervised processes. Finally, the training process of GAN is not considered an easy task and generally takes a lot of time, which might require recording longer initial datasets to start with [86, 103]. Moreover, GANs could be more complex than other data augmentation techniques because of their adversarial nature.

Ultimately, future studies could provide a better insight into why augmentation with GANs improves the classification accuracy in various applications. This aligns with the goals of developing explainable artificial intelligence techniques in the domain of EEG analysis [104]. Another direction to explore is using GANs to augment limited training data, which could result in reducing the amount of calibration data needed for practical EEG-based applications. This idea could be extended to additionally reduce the testing data, especially in applications that require recording multiple trials for the same task. Moreover, GANs could be utilized in developing across-subject models which have potential applications in transfer learning and in online BCI designs. Finally, optimal channel selection and the selection of the best set of hyperparameters for GANs require more investigations in order to avoid the high computational load and the generation of redundant data.

## Conclusion

Undeniably, the small size of EEG datasets represents a challenge when being analyzed using machine and deep learning techniques. There are various reasons for the scarcity of EEG data including the availability of subjects, session time, and procedure complexity. GAN, as a current rising deep learning technique, showed outstanding results in augmenting data in different fields including images, and video. Furthermore, it has proven to be a promising approach to improving the performance of DNNs. Thus, in this article, we provided a comprehensive overview of state-of-the-art GAN methods applied to EEG data. To our knowledge, this is the first article that focuses on using GANs in different applications involving EEG signals. Based on this overview, we could conclude that GANs are able to successfully improve performance in different EEG-based applications. Further investigations should be conducted to address various issues associated with using GANs in this field.

## Data Availability

All data is available either as part of the article or as additional files.
